# SNP-based mate allocation strategies to maximize total genetic value in pigs

**DOI:** 10.1186/s12711-019-0498-y

**Published:** 2019-09-27

**Authors:** David González-Diéguez, Llibertat Tusell, Céline Carillier-Jacquin, Alban Bouquet, Zulma G. Vitezica

**Affiliations:** 1 GenPhySE, INRA, Université de Toulouse, 31326 Castanet-Tolosan, France; 2 IFIP Institut du Porc, BP35104, 35651 Le Rheu, France; 3 France Génétique Porc, BP35104, 35651 Le Rheu, France

## Abstract

**Background:**

Mate allocation strategies that account for non-additive genetic effects can be used to maximize the overall genetic merit of future offspring. Accounting for dominance effects in genetic evaluations is easier in a genomic context, than in a classical pedigree-based context because the combinations of alleles at loci are known. The objective of our study was two-fold. First, dominance variance components were estimated for age at 100 kg (AGE), backfat depth (BD) at 140 days, and for average piglet weight at birth within litter (APWL). Second, the efficiency of mate allocation strategies that account for dominance and inbreeding depression to maximize the overall genetic merit of future offspring was explored.

**Results:**

Genetic variance components were estimated using genomic models that included inbreeding depression with and without non-additive genetic effects (dominance). Models that included dominance effects did not fit the data better than the genomic additive model. Estimates of dominance variances, expressed as a percentage of additive genetic variance, were 20, 11, and 12% for AGE, BD, and APWL, respectively. Estimates of additive and dominance single nucleotide polymorphism effects were retrieved from the genetic variance component estimates and used to predict the outcome of matings in terms of total genetic and breeding values. Maximizing total genetic values instead of breeding values in matings gave the progeny an average advantage of − 0.79 days, − 0.04 mm, and 11.3 g for AGE, BD and APWL, respectively, but slightly reduced the expected additive genetic gain, e.g. by 1.8% for AGE.

**Conclusions:**

Genomic mate allocation accounting for non-additive genetic effects is a feasible and potential strategy to improve the performance of the offspring without dramatically compromising additive genetic gain.

## Background

Mate allocation has been used in animal breeding schemes mainly to control inbreeding but also to increase connectedness among herds, to preserve genetic diversity, and to exploit dominance [1–4]. The total genetic merit of future progeny can be maximized by selecting pairs of mates to better exploit dominance, especially in crossbreeding programs (across breeds), but also within a population [5, 6].

In the classical pedigree context, obtaining accurate estimates of dominance effects is difficult because it requires large groups of individuals with dominance relationships (the probability of identical genotypes of two individuals at a locus), such as full-sibs, and also because estimations are computationally demanding [7, 8]. With the availability of high-density single nucleotide polymorphism (SNP) panels and large numbers of genotyped animals, genomic selection has improved the efficiency of animal breeding programs, essentially by reducing generation intervals and improving prediction accuracies of breeding values of difficult-to-measure and lowly heritable traits [9].

Accounting for dominance effects in a GBLUP (genomic best linear unbiased prediction) framework is easier than in a classical pedigree-based context because, instead of probabilities of identical genotypes, we have observations of heterozygous states at SNPs across the genome [10]. Thus, the availability of genomic information has renewed interest in non-additive genetic effects. Genomic models that include dominance have been implemented in several livestock populations, including dairy cattle, pigs, sheep, and layer chickens [11–17]. The inclusion of dominance in genetic evaluation models has allowed the quantification of the magnitude of non-additive genetic effects and determination of the genetic structure of traits. In general, inclusion of dominance effects in genomic prediction models did not increase the accuracy of estimates of breeding values [18] but may be important for implementing mate allocation strategies to exploit non-additive genetic effects [2, 11, 19].

Mate allocation strategies are based on the idea that, although selection should be based on estimated breeding values (heritable effects), the animals used for commercial purposes can be the product of planned matings that maximize the total genetic merit (additive plus dominance effects) of the offspring [2]. Mate allocation accounting for dominance increased progeny performance in one simulated population [2] and when based on dairy cattle data [11, 19].

In pig breeding schemes, although the goal is to improve crossbreed performance (for commercial production), selection is traditionally performed within purebreds [20, 21]. Pig breeding schemes could benefit from mate allocation strategies and secure additional profit from across- and within-breed dominance variation [6]. In pigs, estimates of dominance variance based on pedigree-based estimation ranged from 11% of the additive genetic variance for backfat to 78% for litter weight at 21 days [22]. The large magnitude of these estimates suggests a potential gain in total genetic merit if matings are allocated to exploit dominance.

The aim of this study was twofold. First, the dominance variance for age at 100 kg, backfat depth at 140 days, and average piglet weight at birth within litter was estimated in Landrace pigs. Second, mate allocation strategies that focus on maximizing either the average breeding value or the average total genetic value for prospective progeny were compared.

## Methods

### Phenotypes and genotypes

Data were provided by a collective French Landrace pig program led by the breeding companies NUCLEUS (Le Rheu, France) and AXIOM (Azay-sur-Indre, France). Animal Care and Use Committee approval was not requested for this study because the data used were routinely collected data according to standard practices in breeding herds. Performances recorded in their selection herds and test stations are centralized to perform common genetic evaluations. The main selection objective of the collective program is the improvement of reproductive criteria. However, improving growth and feed efficiency traits without impairing carcass composition and meat quality is also considered in the breeding goal [23]. The Landrace breed is renowned for its high prolificacy, excellent maternal instinct, and milking abilities. It has also good rusticity, fattening performance, and meat quality and carcass composition characteristics [24]. Data on age at 100 kg (AGE), backfat depth (BD) at 140 days, and average piglet weight at birth within litter (APWL) were analyzed. Performances were recorded in breeding herds at around 100 kg live weight for AGE and BD. Backfat depth was measured ultrasonically at the shoulder, last rib and hip joint on each side of the animal (4 cm from the mid-dorsal spine line) and an average BD was calculated for each animal using these six measurements. AGE and BD were adjusted to a 100 kg live weight; AGE was adjusted to 100 kg based on individual age ($age_{test}$) and weight ($w_{test}$) at the test, and mean age ($age_{batch}$) and weight ($w_{batch}$) of the batch as: $AGE = age_{test} + \left[ {r - 0.0077w_{batch} + 0.0047*age_{batch} } \right]*\left( {100 - w_{test} } \right)$, with $r$ equal to 1.05 and 1.125 for males and females, respectively; BD was adjusted to 100 kg as: $BD = averageBD + r*\left( {100 - w_{test} } \right)$, with $r$ equal to 0.1 and 0.12 for males and females, respectively. APWL was considered as a sow trait and was the average of individual birth weights of piglets. It was recorded for each litter, with an average of 2.7 parities per sow. Only Landrace animals with genotypes were included in the analysis. Average performance and number of animals and records used for analyses of the three traits are in Table 1.

**Table 1:** Numbers of animals, records, and means (SD) for the three evaluated traits

Trait	Number of animals		Number of records	Mean
	Males	Females		
AGE (days)	789	2179	2968	149.0 (9.4)
BD (mm)	1007	2675	3682	11.2 **(**1.7)
APWL (g)	1446	1226	3297^a^	1322 **(**213)

*AGE* age at 100 kg, *BD* backfat depth, *APWL* average piglet weight at birth within litter

^a^Only for females as APWL is a maternal trait with repeated measurements

Pedigrees were extracted from the collective French Landrace pig program and included all available ancestors of the genotyped animals in the datasets. Three generations were traced back from the pedigree for each trait, resulting in 5534, 6541, and 3331 animals for AGE, BD and APWL, respectively. Two medium-density panels were used for genotyping: the porcine SNP60 Illumina BeadChip (Illumina, San Diego, CA) and the GeneSeek Genomic Profiler HD 80 k (GeneSeek, Licoln, NE), on 98% and 2% of the genotyped animals, respectively. SNPs with a call rate lower than 0.98, a minor allele frequency lower than 0.05, and deviating from Hardy–Weinberg equilibrium (*P* value < 0.05) were removed. Six animals with a call rate lower than 0.98 were discarded. Three offspring that displayed Mendelian inconsistencies with their parents were removed. After quality control, 39,353 SNPs that were in common between the two panels were retained for analyses and used to build the genomic relationship matrices.

### Estimation of variance components

Three linear mixed models (one pedigree-based and two genomic-based models) were used to estimate variance components for each trait. The first model (model A) was a classical additive genetic model in which the genetic relationship matrix was computed based on pedigree information from genotyped animals (a sample of the French Landrace population). The genomic models included either additive genetic effects (model G) or additive and dominance genetic effects (model GD). The two genomic models included a genomic inbreeding depression parameter and were implemented using a GBLUP framework. Fixed effects for AGE were the combination of farm-year-sex (68 levels) and the covariate of birth weight. Fixed effects for BD included the combination of farm-year-sex (90 levels), parity of the dam (6 levels), and litter size as a covariate. The APWL trait included farrowing batch (283 levels) and parity of the dam (6 levels) as fixed effects and an animal permanent environmental random effect (1226 sows).

In matrix notation, model G can be represented as follows:

$${\mathbf{y}} = {\mathbf{X\varvec{\upbeta} }} + {\mathbf{f}}{\text{b}} + {\mathbf{Zu}} + {\mathbf{Zpe}} + {\mathbf{e}}\text{,}$$

where ${\mathbf{y}}$ is the vector of phenotypes of a trait; ${\varvec{\upbeta}}$ is the vector of fixed effects; ${\mathbf{f}}$ is the vector of genomic inbreeding coefficients, calculated as the proportion of homozygous SNP genotypes for each animal, and ${\text{b}}$ is the inbreeding depression parameter; ${\mathbf{u}}$ is the vector of additive genetic effects (i.e. breeding values); ${\mathbf{pe}}$ is the vector of random permanent environmental effects specific to APWL $\left( {{\mathbf{pe}}\sim N\left( {0,\varvec{ }{\mathbf{I}}\sigma_{pe}^{2} } \right)} \right)$; and ${\mathbf{e}}$ is the vector of residual effects $\left( {{\mathbf{e}}\sim N\left( {0, {\mathbf{I}}\sigma_{e}^{2} } \right)} \right)$. Breeding values were assumed distributed as ${\mathbf{u}}\sim N\left( {0,{\mathbf{G}}\sigma_{A}^{2} } \right)$, where ${\mathbf{G}}$ is the additive genomic relationship matrix (model G). The incidence matrix ${\mathbf{X}}$ relates observations to fixed effects, and ${\mathbf{Z}}$ is the incidence matrix for breeding values and permanent environmental random effects. Model A did not include the inbreeding depression parameter and breeding values were assumed distributed as ${\mathbf{u}}\sim N\left( {0,{\mathbf{A}}\sigma_{A}^{2} } \right)$, where ${\mathbf{A}}$ is the additive pedigree-based relationship matrix. Parameters $\sigma_{pe}^{2}$, $\sigma_{A}^{2}$, and $\sigma_{e}^{2}$ refer to permanent environmental, additive genetic, and residual variances, respectively.

The additive genomic relationship matrix was calculated according to VanRaden [25] as:

$${\mathbf{G}} = \frac{{{\mathbf{MM^{\prime}}}}}{{2\mathop \sum \nolimits_{k = 1}^{m} p_{k} q_{k} }},$$

where ${\mathbf{M}}$ is a matrix with dimensions of number of animals $\left( n \right)$ by number of SNPs $\left( m \right)$, with elements equal to $\left( {2 - 2p_{k} } \right)$, $\left( {1 - 2p_{k} } \right)$ and $- 2p_{k}$, for genotypes $AA$, $Aa$ and $aa$ respectively; $p_{k}$ is the frequency of allele $A$ of the $k$^th^ SNP (with $k = 1, \ldots ,m$), and $q_{k} = 1 - p_{k}$. Elements of ${\mathbf{M}}$ for missing SNP genotypes were set to the average of the population.

Model GD is an expansion of model G, which takes dominance genetic effects into account:

$${\mathbf{y}} = {\mathbf{X\varvec{\upbeta} }} + {\mathbf{f}}{\text{b}} + {\mathbf{Zu}} + {\mathbf{Zv}} + {\mathbf{Zpe}} + {\mathbf{e}},$$

where ${\mathbf{v}}$ is the vector of random dominance deviation effects, assumed distributed as ${\mathbf{v}}\sim N\left( {0,{\mathbf{D}}\sigma_{D}^{2} } \right)$, where $\sigma_{D}^{2}$ is the variance of dominance deviations. The dominance genomic relationship matrix ${\mathbf{D}}$ was built as in Vitezica et al. [10]:

$${\mathbf{D}} = \frac{{{\mathbf{WW^{\prime}}}}}{{\mathop \sum \nolimits_{k = 1}^{m} \left( {2p_{k} q_{k} } \right)^{2} }},$$

where ${\mathbf{W}}$ has the same dimension as ${\mathbf{M}}$, with elements equal to $- 2q_{k}^{2}$, $2p_{k} q_{k}$ and $- 2p_{k}^{2}$ for the $AA$, $Aa$ and $aa$ genotypes, respectively. Elements of ${\mathbf{W}}$ for missing SNP genotypes were set to the average of the population. Matrices ${\mathbf{G}}$ and ${\mathbf{D}}$ were scaled in order to improve numerical stability as ${\mathbf{G}}^{*} = 0.95{\mathbf{G}} + 0.05{\mathbf{I}}$, and ${\mathbf{D}}^{*} = 0.95{\mathbf{D}} + 0.05{\mathbf{I}}$, where ${\mathbf{I}}$ is the identity matrix. Genomic inbreeding was used to account for directional dominance [16, 26] by including it as a covariate in the model to obtain the correct estimate of dominance variance [16, 19].

Variance components for the three models described above were estimated by EM-REML with the *remlf90* software [27], which is available at http://nce.ads.uga.edu/wiki/doku.php. Asymptotic standard errors of variance components and variance proportion estimates were obtained as in Houle and Meyer [28].

The three models were compared using the Akaike Information Criterion (AIC) [29]. The model with the minimum AIC value was considered as the best model to fit the data.

### Estimation of additive and dominance SNP effects

Marker effects were estimated by using a BLUP-SNP model that assumes that the variance components estimated above are known parameters (*GS3* software available at http://snp.toulouse.inra.fr/~alegarra/, Legarra et al. [30]). This marker-level model (in contrast to the previous animal-level models) is represented as follows:

$${\mathbf{y}} = {\mathbf{X\varvec{\upbeta} }} + {\mathbf{f}}{\text{b}} + {\mathbf{Ka}} + {\mathbf{Td}}^{*} + {\mathbf{Zpe}} + {\mathbf{e}},$$

where ${\mathbf{a}}$ and ${\mathbf{d}}^{\varvec{*}}$ are random vectors of additive and dominance marker effects, respectively, and all other terms are as described for the animal-level models. Xiang et al. [16] proved analytically that inclusion of genomic inbreeding in genetic models accounts for directional dominance and inbreeding depression. The vector of dominance marker effects was defined as ${\mathbf{d}}^{ *} = {\mathbf{d}} - E\left( {\mathbf{d}} \right) = {\mathbf{d}} - \mu_{d}$, where $\mu_{d} = - \frac{\text{b}}{m}$, which makes $E\left( {{\mathbf{d}}^{*} } \right) = 0$. Incidence matrices ${\mathbf{K}}$ and ${\mathbf{T}}$ relate data to additive and dominance marker effects, respectively, and they are coded as 1, 0 and − 1 for additive effects and as 0, 1 and 0 for dominance effects of the three SNP genotypes $AA$, $Aa$ and $aa$, respectively. Variances of the vectors of additive and dominance effects are $V\left( {\mathbf{a}} \right) = {\mathbf{I}}\sigma_{a}^{2}$ and $V\left( {\mathbf{d}} \right) = {\mathbf{I}}\sigma_{d}^{2}$, where ${\mathbf{I}}$ is the identity matrix, and $\sigma_{a}^{2}$ and $\sigma_{d}^{2}$ are the variances of additive and dominance SNP effects, respectively. Variance components at the marker level ($\sigma_{a}^{2}$ and $\sigma_{d}^{2}$) were obtained from genetic variance components [11] that were estimated by EM-REML with model GD ($\sigma_{A}^{2}$ and $\sigma_{D}^{2}$), as follows:

$$\sigma_{d}^{2} = \frac{{\sigma_{D}^{2} }}{{\sum \left( {4p_{k}^{2} q_{k}^{2} } \right)}},$$

$$\sigma_{a}^{2} = \frac{{\sigma_{A}^{2} - \sum \left[ {2p_{k} q_{k} \left( {q_{k} - p_{k} } \right)^{2} } \right]\sigma_{d}^{2} }}{{\sum \left( {2p_{k} q_{k} } \right)}}.$$

After estimating the SNP effects ${\mathbf{a}}$ and ${\mathbf{d}}^{\varvec{*}}$, estimates of the marker dominance effects were recovered as ${\mathbf{d}} = {\mathbf{d}}^{\varvec{*}} - \frac{\text{b}}{m}$, where ${\text{b}}$ is the inbreeding depression parameter estimated in this model and $m$ is the number of SNPs [16].

### Prediction of progeny total genetic and breeding values

From the estimates of additive and dominance SNP effects, the total genetic value $\left( {g_{ij} } \right)$ of the progeny from a mating between the $i$th boar and $j$th sow, was predicted following Toro and Varona [2] as:

$$\widehat{g}_{ij} = \mathop \sum \limits_{k} \left[ {P_{ijk} \left( {AA} \right)\widehat{a}_{k} + P_{ijk} \left( {Aa} \right)\widehat{d}_{k} + P_{ijk} \left( {aa} \right)( - \widehat{a}_{k} )} \right],$$

where $P_{ijk} \left( {AA} \right)$, $P_{ijk} \left( {Aa} \right)$ and $P_{ijk} \left( {aa} \right)$ are the probabilities of genotypes $AA$, $Aa$ and $aa$ from the progeny of the mating between the $i$th and the $j$th individuals at the $k$th SNP, and $\widehat{a}_{k}$ and $\widehat{d}_{k}$ are the additive and dominance estimated effects of the $k$th SNP.

Analogously, the breeding value $\left( {u_{ij} } \right)$ of the progeny of the same mating was predicted as:

$$\widehat{u}_{ij} = \mathop \sum \limits_{k} \left[ {P_{ijk} \left( {AA} \right)\left( {2 - 2p_{k} } \right)\widehat{\alpha }_{k} + P_{ijk} \left( {Aa} \right)\left( {1 - 2p_{k} } \right)\widehat{\alpha }_{k} + P_{ijk} \left( {aa} \right)\left( { - 2p_{k} } \right)\widehat{\alpha }_{k} } \right],$$

where $\widehat{\alpha }_{k}$ is the allele substitution effect for the $k$th SNP, calculated as $\widehat{\alpha }_{k} = \widehat{a}_{k} + \widehat{d}_{k} \left( {q_{k} - p_{k} } \right)$ [31]. Missing genotypes were ignored and did not contribute to the prediction of progeny values.

### Allocation of matings

The total genetic and the breeding values of the progeny were computed for all potential matings between 120 boars, representing the number of boars that are selected per year, and all available genotyped sows for each trait. In total, 789, 1007 and 1446 males were available for AGE, BD, and APWL (Table 1), respectively, of which the best 120 were selected based on their genomic estimated breeding value (GEBV) based on the estimated SNP effects. For example, for BD, the total number of possible matings was 321,000 (120 boars × 2675 sows). From all possible combinations, the best set of 600 matings was selected based either on the expected additive breeding value of the progeny $\left( {\widehat{u}} \right)$ to maximize additive genetic gain of the expected progeny, equivalent to the traditional selection program for genetic improvement, or on the expected total genetic value of the progeny $\left( {\widehat{g}} \right)$ to maximize total genetic gain of the expected progeny. The task of maximizing additive gain or total genetic gain of progeny was addressed via linear programming [32] using the R [33] *lpsolve* package [34]. Two constraints were set for the optimization: (1) each boar could be mated to up to 15 sows and (2) each sow could not be mated to more than one boar. In this setting, optimization was done for both boars and sows. The total number of sows was always equal to the number of selected matings (600), but the number of boars and their contribution to the progeny could differ depending on the mate allocation strategy. As a result, the objective function for the linear program, for instance for the total genetic value $\left( {\widehat{g}_{ij} } \right)$, was defined as:

$$f_{optim} \left( {\widehat{g}_{ij} } \right) = \mathop \sum \limits_{i = 1}^{nb} \mathop \sum \limits_{j = 1}^{ns} \widehat{g}_{ij} x_{ij} ,$$

with constraint $x_{i1} + x_{i2} + x_{i3} + \cdots + x_{is} = 15 \left( {i = 1,2, \ldots ,nb} \right)$ for boar $i$ and: $x_{1j} + x_{2j} + x_{3j} + \cdots + x_{bj} \le 1 \left( {j = 1,2, \ldots ,ns} \right)$ for sow $j$, where $x_{ij}$ are binary decision variables, with 0 representing that the mating between boar $i$ and sow $j$ was not selected, and 1 that the mating was selected; $nb$ and $ns$ correspond to the number of boars (120) and sows (2179, 2675, and 1226 for AGE, BD and APWL (Table 1), respectively). In order to compare the results for these two mate allocation strategies (i.e. based on $\widehat{u}$ or on $\widehat{g}$), differences between the mean $\widehat{u}$ (or the mean $\widehat{g}$) of selected matings and the mean $\widehat{u}$ (or the mean $\widehat{g}$) of all possible matings were calculated. These differences were called expected additive genetic gain ($\Delta U$) and expected total genetic superiority ($\Delta G$).

## Results

### Variance components and heritabilities

Table 2 shows the estimates of variance components, narrow-sense heritabilities and variance ratios that were obtained with the three models for the three traits under investigation. Our estimates of additive and dominance variances are presented in terms of breeding values and dominance deviations [10]. For AGE and BD, estimates of additive genetic variances were similar between the two genomic models (G and GD), regardless of whether non-additive genetic effects (dominance and inbreeding) were included. Estimates of additive genetic variance obtained with model A differed to some extent from those obtained with the genomic models for AGE, and especially for BD. In all cases, standard errors of the estimates of additive variance were higher in model A than in the genomic models. Estimates of heritabilities for AGE and BD were lower when based on the genomic models compared to the pedigree-based model, but these differences were not significant. For APWL, estimates of heritability were consistent across the three models.

**Table 2:** Variance component estimates (standard error) obtained with models A, G and GD

Trait	Model	$\sigma_{A}^{2}$	$\sigma_{D}^{2}$	$\sigma_{pe}^{2}$	$\sigma_{e}^{2}$	$h^{2}$	$\sigma_{D}^{2} /\sigma_{A}^{2}$	$\sigma_{D}^{2} /\sigma_{P}^{2}$
AGE (d)	A	20.92 (3.22)			41.77 (2.42)	0.33 (0.05)		
	G	16.60 (2.18)			45.83 (1.67)	0.27 (0.03)		
	GD	16.26 (2.17)	3.31 (1.72)		42.80 (2.15)	0.26 (0.03)	0.20 (0.11)	0.05 (0.03)
BD (mm)	A	0.52 (0.07)			1.34 (0.06)	0.28 (0.04)		
	G	0.36 (0.05)			1.49 (0.04)	0.19 (0.02)		
	GD	0.36 (0.05)	0.04 (0.04)		1.45 (0.06)	0.19 (0.02)	0.11 (0.11)	0.02 (0.02)
APWL (g)	A	14,503.80 (2218.07)		3665.22 (1593.56)	21,499.64 (696.63)	0.37 (0.05)		
	G	14,476.03 (1860.53)		3793.68 (1128.60)	21,558.88 (694.24)	0.36 (0.04)		
	GD	14,312.09 (1845.51)	1700.24 (1597.04)	2234.86 (1792.05)	21,562.79 (684.33)	0.36 (0.04)	0.12 (0.11)	0.04 (0.04)

*AGE* age at 100 kg, *BD* backfat depth, *APWL* average piglet weight at birth within litter, *A* pedigree-based model including only additive genetic effects, *G* genomic model including additive genetic effects and inbreeding, *GD* genomic model including additive and dominance genetic effects and inbreeding, $\sigma_{A}^{2}$ additive genetic variance, $\sigma_{D}^{2}$ dominance genetic variance, $\sigma_{pe}^{2}$ permanent environmental variance, $\sigma_{e}^{2}$ residual variance, $h^{2}$ heritability, $\sigma_{P}^{2}$ phenotypic variance

The proportion of phenotypic variance explained by dominance genetic effects were 5, 2, and 4% for AGE, BD and APWL, respectively. The influence of dominance genetic effects was greater for AGE than for BD and APWL. In addition, AGE also had the highest ratio of dominance genetic variance to additive genetic variance (0.20), while this ratio was similar for BD and APWL (0.11 and 0.12, respectively). When dominance was included in model GD for analyses of AGE and BD, a small reduction in residual variance was observed for both traits compared to model G. This means that most of the dominance variance was absorbed in the residual variance for these traits in model G. For APWL with model G, where a repeatability model was used, most of the dominance variance was included in the permanent environmental variance.

### Effect of inbreeding

The average proportion of homozygosity or genomic inbreeding per animal was 0.62 (SD = 0.015) in this Landrace population. Estimates of inbreeding depression (b), expressed as the change in phenotypic mean per 10% increase in genomic inbreeding (standard error), were equal to 4.29 (1.17) days, − 0.01 (0.18) mm, and 42.38 (32.66) g for AGE, BD, and APWL, respectively. The impact of inbreeding was negative for AGE (i.e. inbred animals required more days to reach 100 kg), negligible for BD, and positive for APWL, but not different from zero for BD and APWL.

### Goodness of fit

Table 3 shows the AIC value for each model. For all traits, genomic models fitted the data better than the pedigree-based model. The two genomic models had very similar AIC values for BD and APWL. For AGE, which was the trait with the highest ratio of dominance to additive variance, model GD fitted the data slightly better than model G but this improvement can be considered irrelevant.

**Table 3:** Value of the akaike information criterion (AIC) of the fitted models for each trait

Trait	Model		
	A	G	GD
AGE	20,205.4	20,158.1	20,155.6
BD	12,491.7	12,485.8	12,486.7
APWL	40,384.1	40,310.2	40,311.0

*AGE* age at 100 kg, *BD* backfat depth, *APWL* average piglet weight at birth within litter, *A* pedigree-based model including only additive genetic effects, *G* genomic model including additive genetic effects and inbreeding, *GD* genomic model including additive and dominance genetic effects and inbreeding

### Prediction of total genetic values of matings

Selection in pigs aims at reducing age at 100 kg (AGE) and backfat depth (BD), and increasing the average piglet weight at birth within litter (APWL). As expected, when matings were selected on the mean breeding value $\left( {\widehat{u}} \right)$, 40 boars and 600 sows were selected, i.e. those with the best GEBV. Thus, mate selection on $\widehat{u}$ was equivalent to randomly mating males and females that were selected by standard truncation selection on GEBV. When the matings were selected on the mean total genetic value $\left( {\widehat{g}} \right)$, most selected sows were the same as those selected when maximizing $\widehat{u}$, with the number of sows in common between the selection strategies being equal to 560, 583, and 590 out of 600 for AGE, BD, and APWL, respectively. The number of boars selected when maximizing $\widehat{g}$ was equal to 56, 44, and 55 for AGE, BD, and APWL, respectively. This indicates that different boars were contributing when mates were selected to optimize $\widehat{g}$. The top 40 boars selected to maximize $\widehat{u}$ were also selected when maximizing $\widehat{g}$, but they contributed differently (i.e. they were mated to different numbers of sows) and they were mated with different sows. For example, for BD, 38 boars reached the restriction of 15 sows per boar and these boars had the highest GEBV. The remaining six boars were mated to 14, 6, 6, 2, 1, and 1 sows. For AGE and APWL, 32 and 34 boars, respectively, reached the restriction of 15 sows, and these boars had the highest GEBV as well.

The mean $\widehat{u}$ (or $\widehat{g}$) for all possible matings was − 2.02 (− 2.03) days, − 0.35 (− 0.35) mm, and 78.08 (78.08) g for AGE, BD, and APWL, respectively. The difference between the mean $\widehat{u}$ (or $\widehat{g}$) of selected matings and the mean of all matings was called expected additive genetic gain ($\Delta U$) and expected total genetic superiority ($\Delta G$), respectively (Table 4). The expected additive genetic gain and total genetic superiority that were obtained with the selected matings are presented for each mating strategy in Table 4. Note that negative values for AGE and BD are favorable, while a positive value is favorable for APWL. For all traits, the expected total genetic superiority ($\Delta G$ in Table 4) of the progeny was clearly higher when matings were selected based on $\widehat{g}$ compared to on $\widehat{u}$, giving the offspring an advantage of − 0.79 days, − 0.04 mm, and 11.34 g, for AGE, BD, and APWL, respectively. These advantages amounted to 0.20, 0.07, and 0.09 genetic standard deviations (SD) for AGE, BD, and APWL, respectively. The expected additive genetic gains ($\Delta U$ in Table 4) were very similar when matings were selected on $\widehat{u}$ or on $\widehat{g}$ for BD (− 0.58 SD) and APWL (0.49 SD), but was slightly lower (1.8%) for AGE when matings were selected on total genetic value (− 0.56 SD vs − 0.55 SD). This indicates that selecting the candidates to maximize total genetic merit of progeny did not have a detrimental effect on the expected additive genetic gain for these traits.

**Table 4:** Expected total ($\Delta G$) and additive $\left( {\Delta U} \right)$ genetic gain obtained from matings selected on estimated breeding value $\left( {\widehat{u}} \right)$ or on estimated total genetic value $\left( {\widehat{g}} \right)$

Trait	Expected gain	Selection on $\widehat{u}$		Selection on $\widehat{g}$	
		Trait units	Genetic SD units	Trait units	Genetic SD units
AGE (days)	*∆U*	− 2.24	− 0.56	− 2.21	− 0.55
	*∆G*	− 2.19	− 0.54	− 2.98	− 0.74
BD (mm)	*∆U*	− 0.35	− 0.58	− 0.35	− 0.58
	*∆G*	− 0.35	− 0.58	− 0.39	− 0.65
APWL (g)	*∆U*	59.04	0.49	58.35	0.49
	*∆G*	59.44	0.50	70.78	0.59

$\Delta G$ (or $\Delta U$) is the difference between the mean $\widehat{u}$ (or the mean $\widehat{g}$) of selected matings and the mean $\widehat{u}$ (or the mean $\widehat{g}$) of all possible matings of the expected progeny

*AGE* age at 100 kg, *BD* backfat depth, *APWL* average piglet weight at birth within litter

## Discussion

### Variance components and heritabilities

The consistency of the estimates of additive genetic variance across genomic models (G and GD) is attributed to the orthogonality of the models by using the classical parameterization in terms of breeding values and allele substitution effects proposed by Vitezica et al. [35]. The genomic models resulted in lower estimates of additive genetic variance for AGE and BD than the pedigree-based estimates, although the differences were not relevant for practical purposes. This was also observed in other studies in pigs [13, 36, 37] and in dairy cattle [11]. For instance, estimates of genomic heritabilities were approximately half of those estimated with a pedigree-based model in a Duroc population [36] and in a Landrace population [13]. Using different marker densities and sequences, Zang et al. [37] attributed this difference to the so-called “missing heritability” (i.e. incomplete linkage disequilibrium between causal variants and markers), the genetic architecture of the traits and genotype-by-environment interactions. In our study, the main reason for this reduction is that genotyped animals represented a small proportion of the population.

For AGE, reported pedigree-based heritabilities for the French Landrace breed ranged from 0.23 to 0.36 [38–40], which are in agreement with our pedigree-based estimate of 0.33. To our knowledge, genomic estimates of heritability have not been published for Landrace or any other pig breed for this trait. The only comparable genomic estimate refers to lifetime daily gain, which was reported to be 0.27 by Lopes et al. [15] in a Landrace pig population. Pedigree-based estimates of the heritability for backfat reported for the French Landrace breed ranged from 0.46 to 0.55 [38–40] and the only reported genomic estimate in a Landrace population was 0.48 (see File S1 in additional information of Lopes et al. [41]). The heritabilities estimated for BD in our study are lower than those reported in the literature, which may be because the genotyped animals represented a small proportion of the animals in the population and/or because many causal variants for this trait are not in linkage disequilibrium (LD) with the SNPs that were used. Our heritability estimate for APWL was consistent with estimated in previous studies in the same French Landrace population using both genomic and pedigree-based models [42, 43].

Our estimates of the proportion of phenotypic variance explained by dominance effects (Table 2) show that dominance effects contributed only slightly to the phenotypic expression of the traits investigated, and their contributions were lower than the contributions of additive genetic effects. A study in a Yorkshire pig population, using a pedigree-based model that accounted for dominance genetic effects and inbreeding depression, reported proportions of dominance to additive variance of 31 and 11% for days to 104.5 kg and backfat at 104.5 kg, respectively [22]. Our genomic estimate of the proportion of dominance to additive variance for BD (0.11) agrees with this estimate, but our estimate for AGE (20%) was smaller. There are no published genomic estimates of dominance variance for AGE and APWL in any pig breed.

In the literature, estimates of dominance variance are reported based on models with or without inclusion of inbreeding as a covariate. When genomic inbreeding is not included in the model, the dominance variance estimate is inflated, as explained by Xiang et al. [7] and supported by other studies [7, 19, 26]. In our study, the ratio of dominance variance to additive variance was 0.11 for BD but estimates of 0.16 and 0.23 have been reported for backfat [41] using models that ignored inbreeding. This phenomenon has also been observed in cattle [19] for fertility and milk production traits.

In our analyses, estimates of dominance variance were less accurate than estimates of additive variance, which is the case in most studies that estimate dominance effects (e.g. [11, 17, 19]) possibly because of the low magnitude of this variance component, the reduced amount of data available, and the effect of the genotyping strategy. For instance, if only one or two piglets from each litter are genotyped, the proportion of full-sibs is small and, therefore, little dominance-specific information is available for the estimation [11, 44]. A larger number of genotyped individuals per litter allows the detection of identical genotypes among individuals and dominance relationships.

The observed reduction of the permanent environmental variance when dominance was included in the genomic model GD, suggests that a large part of the dominance variance is confounded with the permanent environmental effects when dominance genetic effects are not accounted for. Similar results have also been observed in other studies in cattle [12, 19]. The dominance variance can also be confounded with common-litter effect which was not included in the model as fixed effect.

### Inbreeding effect

The average, across animals, of the proportion of homozygosity or genomic inbreeding in our Landrace population was similar to that (0.69, SD = 0.019) reported by Xiang et al. [16] for a Danish Landrace pig population. Genomic estimates of the effect of inbreeding for these traits are scarce in the literature. One study in a Yorkshire pig population based on a pedigree model reported estimates of inbreeding depression of around 2.10 (days) and 0.00 (mm) per 10% inbreeding, for days to 104.5 kg and backfat at 104.5 kg, respectively [22]. Our results agree with the values reported in the literature for AGE and BD. Our estimate of the effect of inbreeding depression was positive for APWL, but not significantly different from zero.

### Goodness of fit

Genomic models fitted the data better than the pedigree-based model, probably because realized relationships among individuals are better captured by marker information. This is in agreement with previous findings (see e.g. de Los Campos et al. [45]). Differences in AIC between genomic models were small, which means that the inclusion of dominance effects did not significantly improve the goodness of fit of the data. This was in part because model G already included the inbreeding depression parameter, which improved the goodness of fit in a previous study [19]. Aliloo et al. [19] also did not find a difference in goodness of fit between an additive genomic model with average heterozygosity (or inbreeding) and a model that accounted for additive and dominance genetic effects with average heterozygosity.

### Prediction of the total genetic value of matings

Our results confirm that within-breed selection on $\widehat{g}$ profits from dominance effects to maximize the total genetic value of the progeny (productive performance) without compromising the additive genetic gain (based on breeding values). These results are important since the additive genetic value is the genetic component that can be accumulated and inherited by subsequent generations. The negligible reduction in additive genetic gain can be attributed to the fact that almost the same sows and the best boars (based on GEBV) were selected in the matings to optimize $\widehat{g}$ or $\widehat{u}$. In addition, the number of selected boars was larger when selecting on $\widehat{g}$ than when selecting on $\widehat{u}$, which could result in a reduction in the overall inbreeding of the population. Nevertheless, the part of the total genetic gain that is attributable to non-additive genetic effects obtained from mate allocation disappears in subsequent generations, as demonstrated in a simulation by Toro and Varona [2], because non-additive genetic effects depend on the specific allelic combinations that are present between the parents involved in a mating.

Other studies on mate allocation accounting for non-additive genetic effects have also reported increases in total genetic merit compared to selecting on estimated breeding values only. Using simulation, Toro and Varona [2] showed that mate allocation provides an additional selection response in expected progeny of up to 22% (assuming a narrow-sense heritability of 0.20 and a ratio of dominance to phenotypic variance of 0.10) over random mating. In dairy cattle, Sun et al. [46] reported an increase in total genetic gain for milk yield of 9.8 and 7.6% for Holstein and Jersey cows, respectively, from doing mating allocation. Ertl et al. [11] obtained an increase of 14.8 (milk yield) and 27.8% (protein yield) in expected total genetic superiority when matings were selected on $\widehat{g}$ instead of $\widehat{u}$, and, as in our results, only a slight reduction of 4.5% and 2.6% on additive genetic gain was observed. Aliloo et al. [19] reported increases in expected total genetic superiority of 27, 25, and 22% for milk, fat, and protein yield, respectively, in a Holstein population. Our results agree with those reported in the literature in the sense that a greater expected total genetic superiority can be obtained by including non-additive genetic effects to exploit dominance, with a slight or negligible reduction in expected additive genetic gain (1.8% for AGE and 0% for BD and APWL). The largest increase in total genetic gain was observed for AGE (0.20 genetic standard deviations), which had the highest ratio of dominance to additive genetic variance (20%). This greater increase in total genetic gain with a higher ratio of dominance variance was also observed in previous studies that used mate allocation [2, 11], except in [19].

Different mating design scenarios can be found in the literature. Ertl et al. [11] and Aliloo et al. [19] performed a pre-selection of bulls based on their estimated breeding values and only the selection and allocation of females were optimized based on the expected total genetic value of the offspring. This strategy minimizes loss of additive genetic gain since selection intensity is higher for males than for females. Nonetheless, pre-selection of bulls may preclude the opportunity to select other bulls that can potentially produce progeny with a higher total genetic merit when used in specific matings. In our study, optimization on $\widehat{g}$ was made for both males and females, which provides more opportunity to choose matings that have a high total genetic merit in the progeny.

To rule out the possibility that preselection of males based on the GEBV influenced our results, 120 boars were chosen at random and included in the optimization process to select the best set of 600 matings for each mating strategy. Optimization based on $\widehat{u}$ and $\widehat{g}$ yielded similar responses in $\Delta U$ (i.e. − 2.93 vs. − 2.91) but optimization based on $\widehat{g}$ resulted in a higher $\Delta G$ than optimization based on $\widehat{u}$ (i.e. − 3.70 vs. − 2.90). These results confirm that the optimization of the offspring based on $\widehat{g}$ is feasible without reducing $\Delta U$, regardless of the preselection of males.

Our results show that mate allocation strategies for boosting total genetic gain could benefit production levels in the sow herds without placing a cost on additive genetic progress. However, its implementation on a large scale would require some organizational changes, for instance, additional genotyping when not all females are genotyped and high computational costs. An alternative to additional genotyping is to take the maternal grand-sire SNP information into account, as was proposed by DeStefano and Hoeschele [5].

As stated above, genetic gain that is attributable to non-additive genetic effects is expressed in the immediate offspring and is not inherited in subsequent generations. Thus, selection and mating of parents that produce crossbred commercial animals are expected to benefit most from mating allocation strategies for improving total genetic gain in pig production schemes. We focused on single-trait selection but the use of mate allocation strategies can be extended to multiple traits by using an index as objective function to improve total genetic gain as in [19]. The feasibility and the success of mate allocation strategies in a multiple trait approach will depend highly on the dominance genetic correlation between the target traits. Hence, dominance genetic correlations different from 1 will indicate that matings will perform differently between traits. If this is the case, this will inevitably complicate the mating allocation strategy and a compromise between the expected total genetic gains of the traits will be needed.

## Conclusions

In this study, we estimated non-additive genetic variance components and addressed their use to improve the performance of offspring of selected parents by means of mate allocation strategies. Inclusion of non-additive genetic effects is straightforward in a genomic prediction context. For the traits analyzed here, estimates of dominance genetic variance were small, ranging from 11 to 20% of the additive genetic variance. Addition of dominance genetic effects to a model that included additive genetic effects and genomic inbreeding did not improve the goodness of fit of the model. Inbreeding depression was estimated to have an undesirable effect on AGE and no effect on other traits. Genomic mate allocation improved the performance of future offspring of − 0.79 days, − 0.04 mm, and 11.3 g for AGE, BD and APWL, respectively, but slightly reduced the expected additive genetic gain, e.g. by 1.8% for AGE. Our results show that genomic mate allocation, accounting for non-additive genetic effects, is a feasible and a potential strategy to improve the productive performance (total genetic value) of future offspring without compromising the additive genetic gain. Our conclusions are limited to a single population, to a few tested scenarios of mate allocation strategies and under a single-trait approach. Further research is very much needed to explore the benefits of mate allocation strategies in a broader context.

## Data Availability

The database used and analyzed in this study was provided by the national collective breeding scheme of French Landrace pig line and is not available. It comes from private companies and its privacy could be compromised.
